# The effect of postmastectomy radiotherapy in node-positive triple-negative breast cancer

**DOI:** 10.1186/s12885-020-07639-x

**Published:** 2020-11-25

**Authors:** Lei Zhang, Ru Tang, Jia-Peng Deng, Wen-Wen Zhang, Huan-Xin Lin, San-Gang Wu, Zhen-Yu He

**Affiliations:** 1 Sun Yat-sen University Cancer Center, State Key Laboratory of Oncology in South China, Collaborative Innovation Center for Cancer Medicine, 651 Dongfeng Road East, Guangzhou, 510060 China; 2 Department of Radiotherapy, Sun Yat-sen University Cancer Center, 651 Dongfeng Road East, Guangzhou, 510060 People’s Republic of China; 3 Department of Radiation Oncology, Cancer Hospital, the First Affiliated Hospital of Xiamen University, Teaching Hospital of Fujian Medical University, Xiamen, 361003 China

**Keywords:** Breast cancer, Radiotherapy treatment, Prognosis, Survival

## Abstract

**Background:**

The value of postmastectomy radiotherapy (PMRT) for pathological node-positive triple-negative breast cancers (TNBC) remains debatable. The aim of this population-based retrospective study was to evaluate the effect of PMRT on survival outcomes in this population.

**Methods:**

Patients diagnosed with stage T1-4N1-N3M0 TNBC between 2010 and 2014 were identified from the Surveillance, Epidemiology, and End Results (SEER) database. We used univariate and multivariate Cox regression hazards method to determine the independent prognostic factors associated with 3-year breast cancer-specific survival (BCSS). The effect of PMRT on 3-year BCSS was analyzed after stratification by pathological staging of groups.

**Results:**

Of the 4398 patients included in this study, 2649 (60.2%) received PMRT. Younger age, black ethnicity, and advanced tumor (T) and nodal (N) stage were the independent predictors associated with PMRT receipt (all *P* < 0.05). Patients who received PMRT showed better 3-year BCSS (OR = 0.720, 95% CI = 0.642–0.808, *P* < 0.001) than those that did not. The effect of PMRT on 3-year BCSS was analyzed after stratification by pathological staging of groups. The results showed that PMRT was associated with better 3-year BCSS in patients with stage T3–4N1 (*P* = 0.042), T1-4N2 (*P* < 0.001), and T1-4N3 (*P* < 0.001), while comparable 3-year BCSS was found between the PMRT and non-PMRT cohorts with T1–2N1 disease (*P* = 0.191).

**Conclusions:**

Radiotherapy achieved better 3-year BCSS in TNBC patients with stage T3–4N1 and T1-4N2–3 disease. However, no survival benefit was found with the addition of PMRT in patients with T1–2N1 TNBC.

## Background

Breast cancer is the most common malignant cancer and the primary cause of cancer-related mortality in women. Every year, almost 2,76,000 women are newly diagnosed with breast cancer and about 42,000 women die from it in the United States alone [1]. Enhancing survival outcome by multidisciplinary comprehensive treatment remains the global focus of breast cancer. In current clinical practice, the treatment of breast cancer is based not only on the T and N stages of patients but also on the status of estrogen receptor (ER), progesterone receptor, and human epidermal growth factor receptor-2 (HER-2). Breast cancer subtypes based on ER, PR, and HER-2 have been widely used. Triple-negative breast cancer (TNBC) is a subtype characterized by an absence of the ER, PR, and HER-2 status, and accounts for 15% of all breast cancers [2]. TNBC has a higher risk of early metastasis, local recurrence, and poorer prognosis than other types of breast cancer [3].

Mastectomy is one of the local operations for breast cancer. About one-third of all stage I or II and 68% of stage III breast cancer patients undergo mastectomy [3]. The National Comprehensive Cancer Network (NCCN) guidelines recommend radiotherapy for patients with more than four positive lymph nodes, while patients with 1–3 lymph nodes are recommended to receive radiotherapy [4]. However, the recommendation of PMRT does not refer to molecular subtypes of breast cancer. A meta-analysis of 22 randomized trials from the Early Breast Cancer Triallists’ Collaboration Group (EBCTCG) and other studies showed that radiotherapy could obviously decrease recurrence and mortality risk of TNBC and improve overall survival (OS) of patients with positive lymph nodes [5–7]. The value of PMRT for TNBC patients in different pathological nodal stages has not yet been established. The Danish Breast Cancer Cooperative Group (DBCG) and some other studies have shown that there was no significant survival benefit for TNBC patients with positive lymph nodes from receiving PMRT [8–10]. However, insufficient chemotherapy and axillary lymphadenectomy may have likely limited the power of the analysis. A previously conducted retrospective analysis reported no survival improvement with PMRT in patients with T1–2N1 disease [11]. Collectively, previous studies have shown contradictory results in the investigation of survival according to the status of PMRT receipt in different pathological stages of TNBC.

At present, the recommendation of PMRT is mainly based on the pathological T and N stage; the value of PMRT on TNBC patients needs to be further clarified. Therefore, we aimed to identify the effect of PMRT on breast cancer-specific survival (BCSS) in TNBC patients with different pathological nodal stages using the data from the Surveillance, Epidemiology, and End Results (SEER) database.

## Methods

### Study design and patients

All patient information was collected from the SEER database including 18 cancer registries, which represents 28% of the population of the United States (https://seer.cancer.gov/). As this database is open-source and informed consent of patients is not required, ethics committee approval by the institutional review board was not needed for this study. We retrospectively reviewed and analyzed the recorded data of eligible patients in terms of their survival time and BCSS. The following inclusion criteria were applied: female sex, year of diagnosis between 2010 and 2014, diagnosed with pathological T1-4N1–3M0 TNBC, and underwent mastectomy and chemotherapy with or without PMRT. The exclusion criteria were as follows: patients with no information of ethnicity, pathological grade, ER, PR, and HER2 status, and no histology results, and those that received non-external beam radiotherapy or radiotherapy before mastectomy.

### Measures

The tumor sizes and nodal stage were based on the seventh edition of the American Joint Committee on Cancer (AJCC) tumor-staging criteria. We defined the number of months from the date of initial diagnosis to the date of breast cancer-specific death or the date of the last follow-up as BCSS. Age was divided into two groups of < 50 and ≥ 50 years. Ethnicity included white, black, and other (American Indian/AK Native, Asian/Pacific Islander). All patients were divided into four groups according to different pathological stages, namely T1–2N1, T3–4N1, T1-4N2, and T1-4N3 for stratified analysis of the effect of PMRT on TNBC.

### Statistical analysis

The demographic and clinical characteristics of patients were compared using chi-squared test. Prognostic factors of PMRT with odds ratio (OR) and 95% confidence intervals (95% CIs) for BCSS were analyzed by univariate and multivariate Cox regression hazard model. Considering that an increase in follow-up time would lead to a decrease in the number of patients being followed-up [12], the number of patients with > 5-year BCSS in some subgroups enrolled in this study were small (*N* < 20), and some of the selected patients tended to have a short follow-up period which may have led to a bias. Hence, we only used the 3-year BCSS to analyze the prognostic survival value. We considered age, ethnicity, grade, histology, TNM stage, and PMRT as confounding factors for the prognostic analysis; the confounding factors that significantly affected prognosis (*P* < 0.05) were considered as prognostic factors. The BCSS comparisons between PMRT and non-PMRT subgroups were analyzed by log-rank tests and Kaplan–Meier plots. All potential significant prognostic factors (*P* ≤ 0.05) in the univariate analysis were included in multivariable models to adjust analyses, and multivariate analysis was performed to detect the value of PMRT in terms of BCSS. IBM SPSS version 26.0 (IBM Corp., Armonk, NY, USA) was used for all statistical analyses. *P* < 0.05 was considered to indicate statistically significant differences in two-tailed tests.

## Results

### Patient characteristics

A total of 4398 eligible patients were included in this study. All patient characteristics are described in Table 1. The distribution of histology and grade were similar between the PMRT and non-PMRT groups. Based on the staging groups, pathological T1–2N1 stage constituted a high proportion with 46.7% (*n* = 2055) patients.

**Table 1:** Patients’ baseline characteristics of the study population (*n* = 4398)

Variables	N (%)	PMRT (%)	No PMRT (%)	***P*** value
Median follow-up (months)	41.4 (1–83)	41.7 (5–83)	40.8 (1–83)	
Age (years)				
< 50	1713 (38.9)	1098 (41.4)	615 (35.2)	< 0.001
≥ 50	2685 (61.1)	1551 (58.6)	1134 (64.8)	
Ethnicity				
White	3097 (70.4)	1848 (69.8)	1249 (71.4)	0.044
Black	948 (21.6)	601 (22.7)	347 (19.8)	
Other	353 (8.0)	200 (7.6)	153 (8.7)	
Grade				
Well-differentiated	25 (0.6)	13 (0.5)	12 (0.7)	0.318
Moderately differentiated	609 (13.8)	353 (13.3)	256 (14.6)	
Poorly/undifferentiated	3764 (85.6)	2283 (86.2)	1481 (84.7)	
Histology				
IDC^1^	4036 (91.8)	2443 (92.2)	1593 (91.1)	0.314
ILC^2^	71 (1.6)	43 (1.6)	28 (1.6)	
Other	291 (6.6)	163 (6.2)	128 (7.3)	
T3 stage				
T1	883 (20.1)	420 (15.9)	463 (26.5)	< 0.001
T2	2070 (47.1)	1237 (46.7)	833 (47.6)	
T3	833 (18.9)	558 (21.1)	275 (15.7)	
T4	612 (13.9)	434 (16.4)	178 (10.2)	
N4 stage				
N1	2812 (63.9)	1534 (57.9)	1278 (73.1)	< 0.001
N2	937 (21.3)	648 (24.5)	289 (16.5)	
N3	649 (14.8)	467 (17.6)	182 (10.4)	
Staging subgroup				
T1–2N1	2055 (46.7)	1007 (38.0)	1048 (59.9)	< 0.001
T3–4N1	757 (17.2)	527 (19.9)	230 (13.2)	
T1-4N2	937 (21.3)	648 (24.5)	289 (16.5)	
T1-4N3	649 (14.8)	467 (17.6)	182 (10.4)	

1. IDC, invasive ductal carcinoma; 2. ILC, invasive lobular carcinoma; 3. T, tumor; 4. N, nodal

As shown in Table 1, 2649 (60.2%) patients received PMRT (PMRT group), while the remaining 1749 (39.8%) did not (non-PMRT group). In the PMRT group, age < 50 years (41.4% vs. 35.2%), black ethnicity (22.7% vs. 19.8%), and T3 (21.1% vs. 15.7%) and T4 (16.4% vs. 10.2%) stages showed a higher proportion of acceptance rate, while T1 (15.9% vs. 26.5%) and T2 (46.7% vs. 47.6%) stages showed a lower proportion of acceptance rate than in the non-PMRT group. The non-PMRT subgroup presented a significantly higher proportion of pathological N1 stage (73.1% vs. 57.9%) and a lower proportion of pathological N2 (16.5% vs. 24.5%) and N3 (10.4% vs. 17.6%) stages than the PMRT group. Notable differences were detected in pathological T1–2N1 stage between the PMRT and non-PMRT group (38.0% vs. 59.9%), and pathological T3–4N1, T1-4N2, and T1-4N3 stages were more likely to receive PMRT (19.9% vs. 13.2, 24.5% vs. 16.5, 17.6% vs. 10.4%, respectively).

### Survival and prognostic analysis

The predictive factors associated with BCSS were investigated in the cohort of PMRT and non-PMRT groups of TNBC patients. The detailed results are presented in Table 2. The univariate Cox regression analysis revealed age ≥ 50 years, black ethnicity, and advanced staging groups were associated with worse BCSS in univariate analysis (*P* = 0.001, *P* = 0.007 and *P* < 0.001, respectively). However, grade and histology types had no statistically significant impact on the BCSS of TNBC (Table 2). Moreover, PMRT was an independent prognostic factor related to superior BCSS according to adjusted multivariate Cox regression analysis (OR = 0.685, 95% CI = 0.617–0.760, *P* < 0.001).

**Table 2:** Univariate and multivariate analysis for the prognostic factors of BCSS

Variables	Univariate Cox			Multivariate Cox		
	OR^**1**^	95%CI^**2**^	***P*** value	OR	95%CI	***P*** value
Age (years)						
< 50	1			1		
≥ 50	1.200	1.082–1.332	0.001	1.121	1.009–1.245	0.033
Ethnicity			0.001			0.010
White	1			1		
Black	1.177	1.046–1.324	0.007	1.142	1.015–1.285	0.028
Other	0.808	0.660–0.990	0.039	0.834	0.681–1.022	0.081
Grade			0.085			
Well-differentiated	1					
Moderately differentiated	1.101	0.543–2.233	0.789			
Poorly/undifferentiated	1.295	0.646–2.594	0.467			
Histology			0.073			
IDC^3^	1					
ILC^4^	1.147	0.789–1.668	0.472			
Other	1.230	1.023–1.480	0.028			
T^5^ stage			< 0.001			< 0.001
T1	1			1		
T2	1.516	1.289–1.784	< 0.001	1.423	1.209–1.676	< 0.001
T3	2.831	2.379–3.370	< 0.001	2.201	1.791–2.705	< 0.001
T4	4.027	3.370–4.812	< 0.001	2.900	2.363–3.559	< 0.001
N^6^ stage			< 0.001			< 0.001
N1	1			1		
N2	1.953	1.731–2.204	< 0.001	1.753	1.553–1.980	< 0.001
N3	3.206	2.828–3.634	< 0.001	2.677	2.358–3.039	< 0.001
Staging subgroup			< 0.001			< 0.001
T1–2N1	1			1		
T3–4N1	2.394	2.066–2.773	< 0.001	1.249	1.018–1.533	0.033
T1-4N2	2.618	2.285–3.000	< 0.001	1.942	1.666–2.263	< 0.001
T1-4N3	4.324	3.758–4.975	< 0.001	3.004	2.548–3.542	< 0.001
PMRT						
No PMRT	1			1		
PMRT	0.931	0.841–1.029	0.161	0.685	0.617–0.760	< 0.001

1. *OR* odds ratio; 2. *CI* confidence interval; 3. *IDC* invasive ductal carcinoma; 4. *ILC* invasive lobular carcinoma; 5. *T* tumor; 6. *N* nodal

**Fig. 1:**
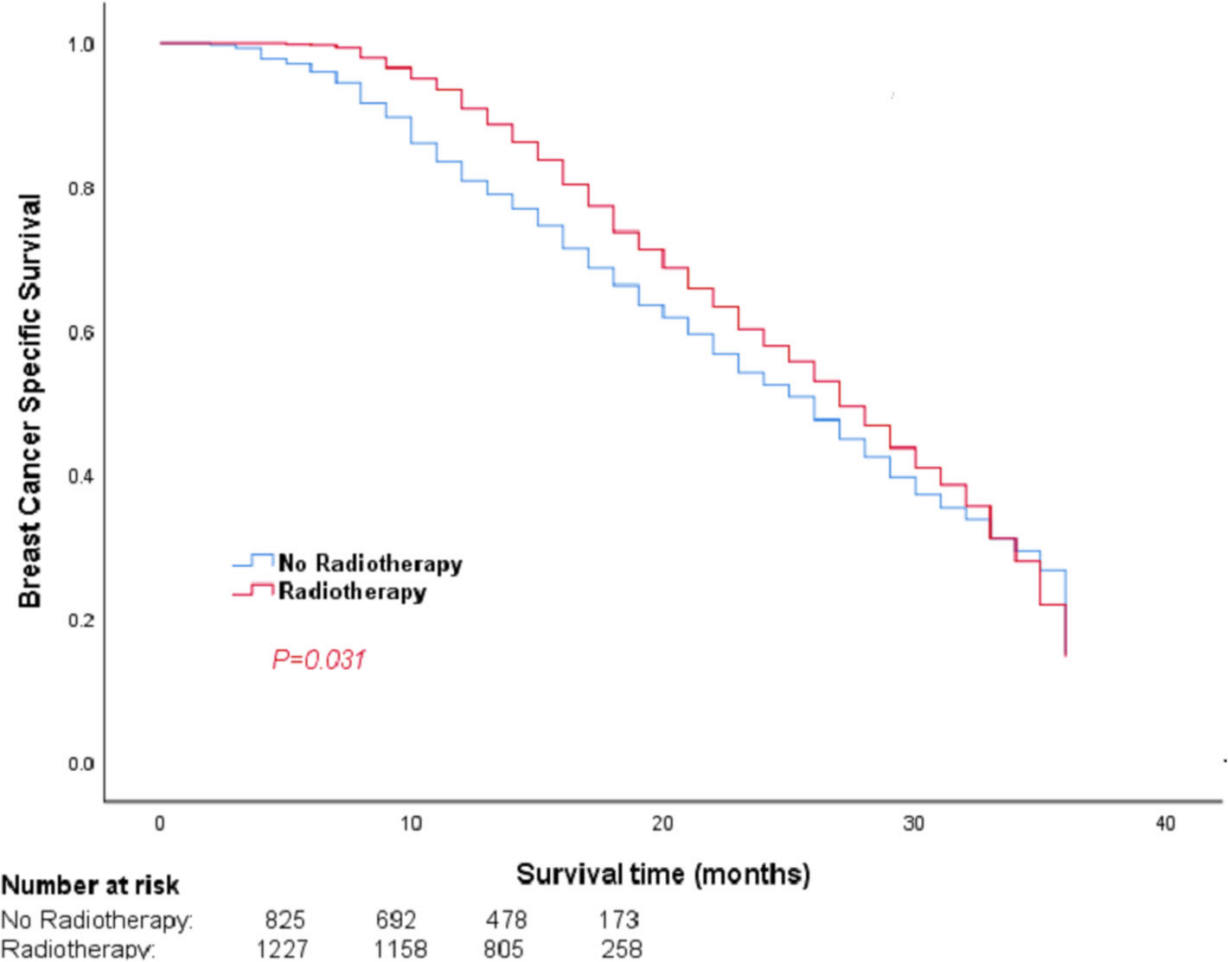
Kaplan–Meier curves and log-rank tests comparing 3-year BCSS for TNBC patients in the overall pathological staging between PMRT and non-PMRT groups

**Fig. 2:**
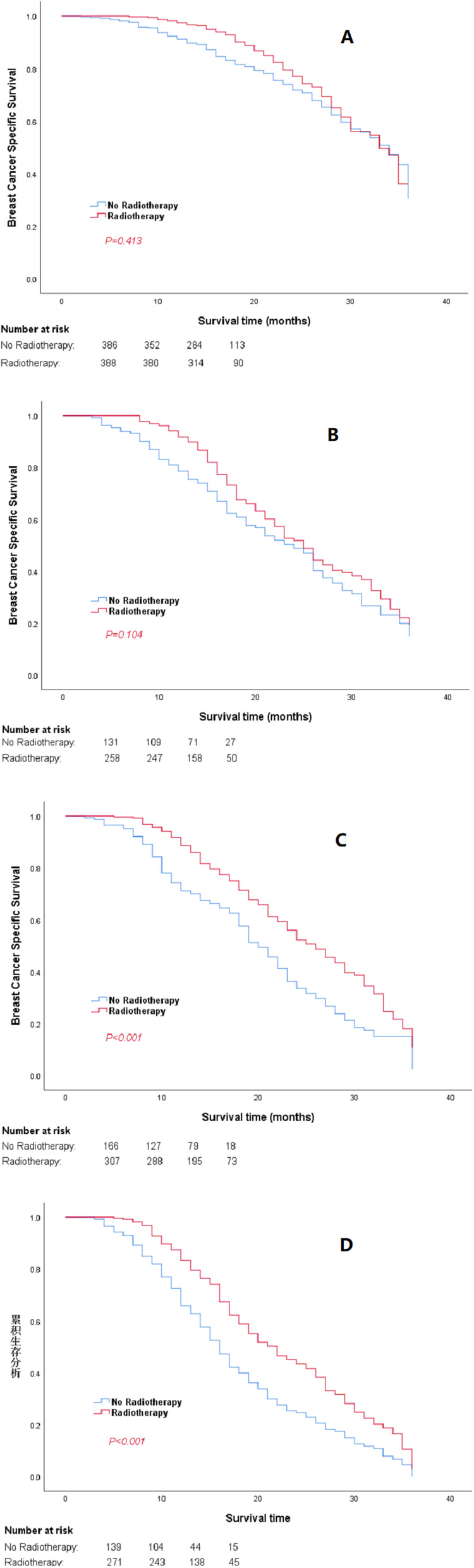
Kaplan–Meier curves and log-rank tests comparing 3-year BCSS for TNBC patients with different staging subgroups between PMRT and non-PMRT groups. **a-d** The 3-year BCSS of patients with T1–2N1, T3–4N1, T1-4N2, and T1-4N3 stage TNBC

With a median follow-up of 41 (range: 1–83) months, a total of 1567 patients (35.6%) died; 73.6% (*n* = 1154) of those patients had breast cancer-related death. The 3-year BCSS was 71.2%, and patients in the PMRT group had better 3-year BCSS than those in the non-PMRT group (*P* = 0.031). However, the prognostic survival analysis showed differences among subgroups. Significantly improved 3-year BCSS was found in the subgroups of patients with T1-4N2 (*P* < 0.001) and T1-4N3 (*P* < 0.001) stages who received PMRT, while patients with pathological T3–4N1 stage TNBC with PMRT had comparable 3-year BCSS with patients that did not receive PMRT (*P* = 0.104). In addition, patients with pathological T1–2N1 stage had no significant difference of 3-year BCSS (*P* = 0.413). The Kaplan–Meier survival curves of 3-year BCSS in overall stages and different staging subgroups are shown in Figs. 1 and 2.

To investigate the effect of PMRT on the 3-year BCSS for TNBC patients in different pathological staging subgroups, multivariate analysis was applied to further analyze the 3-year BCSS stratified by staging subgroups. As shown in Table 3, the results of multivariate analysis indicated that patients who received PMRT in the overall pathological stages had superior 3-year BCSS (*P* < 0.001). Patients with pathological T3–4N1, T1-4N2, and T1-4N3 stages that received PMRT had better survival outcomes than those without PMRT in terms of 3-year BCSS (*P* = 0.042, *P* < 0.001, and *P* < 0.001, respectively). However, patients with pathological T1–2N1 stage showed comparable 3-year BCSS (*P* = 0.191).

**Table 3:** Multivariate Cox proportional hazard analysis evaluating the effect of PMRT stratified by staging subgroups

Staging subgroups (non-PMRT vs. PMRT)	3-year BCSS		
	OR	95% CI (OR)	***P***^***a***^ value
Overall stage	0.720	0.642–0.808	< 0.001
T1–2N1	0.861	0.689–1.078	0.191
T3–4N1	0.765	0.591–0.991	0.042
T1-4N2	0.622	0.501–0.774	< 0.001
T1-4N3	0.630	0.505–0.787	< 0.001

*OR* odds ratio; 2. *CI* confidence interval; 3. *P*^*a*^ value was adjusted by a multivariate Cox proportional hazard regression model including age, ethnicity, pathological T and N stage, and radiotherapy

## Discussion

Although the recommendation for TNBC radiotherapy is similar to that for other breast cancer subtypes, the value of PMRT in TNBC patients remains unclear. In this study, we used a population-based study to retrospectively explore the effect of PMRT on 3-year BCSS among TNBC patients with different pathological stages. Our results showed that PMRT could provide significant benefits to 3-year BCSS for TNBC patients in T1-4N2 and T1-4N3 stages; TNBC patients with pathological T3–4N1 stage disease are more suitable for PMRT considering the results of multivariate analysis have more accuracy than the Kaplan–Meier analysis. However, those with T1–2 stage TNBC showed no benefit from PMRT.

The value of PMRT in patients with pathological T1-T2N1 stage TNBC disease is still controversial. Previous studies have evaluated the efficacy of PMRT on survival outcomes of TNBC patients. The analysis of Kindts et al. showed that TNBC patients that did not receive PMRT had a worse locoregional recurrence rate (LRR) outcome than those that did (HR = 4.45, 95% CI = 1.26–15.69, *P* = 0.020) [13]. Adjuvant radiotherapy after mastectomy has been shown to reduce the 5-year LRR by 17% (6% vs. 23%) and the 15-year breast cancer mortality by 5.4% (54.7% vs. 60.1%, *P* < 0.001) when compared to those without radiotherapy for node-positive cancer [14]. The American Society of Clinical Oncology (ASCO) recommends that PMRT can be used as a standard essential treatment for patients with ≥4 positive axillary lymph nodes [15]; other researchers have accordingly shown that TNBC patients with positive lymph nodes are also equally eligible for PMRT [16, 17]. However, the classification criteria of the number of lymph nodes is difficult to implement for some patients. The beneficial impact of PMRT on disease-free survival (DFS) as reported by Chen et al. supports the use of PMRT in pathological T1-T2N1 stage patients despite the absence of a superior locoregional recurrence-free survival (LRFS) outcome [18]. Recent studies showed that pathological T1–2N1M0 TNBC patients benefit from PMRT with an improved BCSS (*P* = 0.010) [19], while another study in 2004 with 152 TNBC patients showed that pathological T1–2N0–1 stage TNBC without radiotherapy carries a significantly higher risk of LRR (79.6% vs. 57.9%, *P* = 0.049) [20]. The results of the above-mentioned studies were contradictory compared with our outcome, in that pathological T1–2N1M0 stage TNBC patients showed no benefit from PMRT and the 3-year BCSS rates were comparable between the PMRT and non-PMRT subgroups (*P* = 0.191). The inherent selection biases in patient selection and treatment, lack of information about some clinical characteristics, small sample size, and the long interval with respect to the present study are some of the limitations that likely contributed to the inconsistent results.

However, some studies showed that pathological N1 stage TNBC patients do not require PMRT as a necessary treatment [9, 21].. PMRT was associated with an increase in radiation toxicities such as lymphedema, cardiotoxicity, and pneumonitis [22–24]. The consensus of the St. Gallen Breast Cancer Conference showed that more than 64% experts opposed the recommendation of introducing PMRT as a routine treatment for patients with pathological T1–2N1M0 stage breast cancer, and instead recommended considering the omission of PMRT in pathological T1–2 patients with 1–3 positive lymph nodes [25]. Although there are no exact statistics about the efficacy of PMRT in patients with pathological T1–2N1M0 stage TNBC, most clinicians seemed to indicate that PMRT provided no significant benefit for patients with pathological T1–2N1 stage disease in overall breast cancer types. In a retrospective multicenter analysis, Kim et al. found that T1–2N1 stage patients who received PMRT had no obvious improvement of DFS and OS because of the malignant biological features and radio-resistance of TNBC cells [26]. Bhoo-Pathy et al’s study showed that PMRT was not associated with improved survival in T1–2N0–1 TNBC patients, but showed better survival outcome in T3–4N2–3 TNBC patients [27]. The results of these studies are consistent with our outcome. Mastectomy and chemotherapy have been associated with low risk of LRR in TNBC patients with 1–3 positive lymph nodes, and TNBC patients benefit more from chemotherapy when PMRT alone does not provide better curative efficacy [28]. Neo-adjuvant chemotherapy is an important treatment modality in TNBC; however, its effect on PMRT is still controversial and more prospective trials are needed to validate the results [29, 30]. Patients with 1–3 positive lymph nodes who received modern taxane-based chemotherapy had excellent locoregional control; thus, the use of PMRT in patients with 1–3 positive nodes should be tailored to individual patient risks [31]. As seen in our study, PMRT provided no benefits for patients with T1–2N1 stage TNBC. We reasonably assumed that TNBC patients with pathological T1–2N1 stage show relatively good disease control by undergoing mastectomy plus chemotherapy. PMRT is not universally administered in pathological T1–2N1 stage TNBC and should be chosen as a personal requirement especially for patients with higher nodal stages or high-risk biology [32], such as those aged ≥50 years and of black ethnicity as analyzed in our multivariate Cox hazard analysis. Therefore, the risk factors in TNBC patients with pathological T1–2N1 stage who consider receiving PMRT should be fully considered by clinicians.

According to the results of our study, an increasing trend of 3-year BCSS benefit was reflected based on the Kaplan–Meier plots. The patients were stratified for survival analysis by pathological T and N stages for more solid elucidation. Our results validated the data that PMRT is a strong predictive factor of better BCSS for patients with pathological T3–4N1, T1-4N2, and T1-4N3 stage TNBC. Both Kaplan–Meier and multivariate analysis showed that patients with T1-4N2 and T1-4N3 stages who received PMRT had better 3-year BCSS, which was in line with previous studies that reported that additional PMRT for patients with high-risk (stage T3–4 and/or N2–3) TNBC showed superior outcomes in the LRFS and DFS [18, 33]. Patients with pathological T3–4N1 stage TNBC showed contradictory results of 3-year BCSS in both the Kaplan–Meier and multivariate analyses. However, considering that the Kaplan–Meier analysis is a univariate analysis and that the effect of some other factors on PMRT may be omitted, we believe that the results of multivariate analysis are more reliable in order to improve the long-term BCSS. The ASCO recommends considering the need for PMRT for patients with T1–2N1 stage disease by evaluating the risk factors [15]. The NCCN breast cancer guidelines recommend that patients with pathological T3–4 primary tumors or N2–3 axillary lymph nodes after mastectomy should undergo PMRT as a standard adjuvant therapy [4].

Our study has some limitations. First, this is a retrospective analysis from the SEER database rather than a prospective, randomized controlled trial. Therefore, we cannot clarify the reasons for patients choosing PMRT; moreover, the inherent selection biases could undermine the validity of our analysis. Second, some information about patients’ complications, systemic chemotherapy regimens (adjuvant and neo-adjuvant), and the situation of nodal irradiation were absent from the SEER database, which may have led to unconvincing results. At the same time, some of the selected patients in this study had a short follow-up period, and the data might be skewed by the number of patients with relatively short follow-up. Therefore, we did not consider overall survival as an analytical indicator. Thus, we were unable to evaluate the influence of PMRT on LRR of TNBC patients and balance the different possible effects of chemotherapy with PMRT. LRR is an important indicator to evaluate the efficacy of radiotherapy; hence, missing information on LRR, distant metastasis, dose of radiotherapy, and target area are also limitations of the study. Last, SEER may underestimate the reporting rate of radiotherapy. Hence, a randomized controlled clinical study is needed to provide more evidence and a scientific basis to guide clinical treatment. We are currently looking forward to the results of the Selective Use of Postoperative Radiotherapy after Mastectomy trial, where patients with pathological T1–2N1 stage disease were separated randomly into radiotherapy or non-radiotherapy subgroups [34].

## Conclusions

Radiotherapy is an important treatment modality for TNBC patients. The study has provided evidence that patients with pathological T3–4N1 and T1-4N2–3 stage TNBC benefit from PMRT with a better 3-year BCSS; however, PMRT is not beneficial for those with pathological T1–2N1 stage TNBC. These patients should decide whether to undergo PMRT in light of other high-risk factors. Further prospective studies are recommended to elucidate the benefit of PMRT in TNBC patients and provide a strong evidence base for patient selection.

## Data Availability

The authors declare that all datasets generated and analyzed are available in the SEER database (https://seer.cancer.gov/).

## Abbreviations

PMRT: Postmastectomy radiotherapy
TNBC: Triple negative breast cancer
SEER: The Surveillance Epidemiology, and End Results database
BCSS: Breast cancer-specific survival
T: Tumor
N: Nodal
EBCTCG: The Early Breast Cancer Triallists’ Collaboration Group
OS: Overall survival
DBCG: The Danish Breast Cancer Cooperative Group
AJCC: the American Joint Committee on Cancer
OR: Odds ratio
CI: Confidence interval
LRR: Locoregional recurrence rate
ASCO: The American Society of Clinical Oncology
DFS: Disease-free survival
LRFS: Locoregional recurrence-free survival
ALNs: Axillary lymph nodes
